# GLP-1 Receptor Agonists and Gastrointestinal Endoscopy: A Narrative Review of Risks, Management Strategies, and the Need for Clinical Consensus

**DOI:** 10.3390/jcm14155597

**Published:** 2025-08-07

**Authors:** Javier Crespo, Juan Carlos Rodríguez-Duque, Paula Iruzubieta, Eliana C. Morel Cerda, Jose Antonio Velarde-Ruiz Velasco

**Affiliations:** 1 Clinical and Traslational Research in Digestive Diseases, Valdecilla Research Institute (IDIVAL), Faculty of Medicine, University of Cantabria, 39008 Santander, Spain; 2 Clinical and Traslational Research in Digestive Diseases, Valdecilla Research Institute (IDIVAL), Gastroenterology and Hepatology Departments, University Hospital Marqués de Valdecilla, 39008 Santander, Spain; 3 Gastroenterology Department, Hospital Civil de Guadalajara Fray Antonio Alcalde, Guadalajara 44280, Jalisco, Mexico

**Keywords:** GLP-1 receptor agonists, endoscopy, risk

## Abstract

**Background/Objectives**: Glucagon-like peptide-1 receptor agonists (GLP-1 RAs) have transformed the management of type 2 diabetes mellitus and obesity. However, their sustained effect on delaying gastric emptying raises new challenges in gastrointestinal endoscopy performed under sedation. This narrative review aims to summarize current evidence on the impact of GLP-1 RAs on gastric motility and to propose clinical strategies to mitigate associated procedural risks. **Methods**: A narrative review was conducted integrating findings from scintigraphy, capsule endoscopy, gastric ultrasound, and existing clinical guidelines. Emphasis was placed on studies reporting residual gastric content (RGC), anesthetic safety outcomes, and procedural feasibility in patients undergoing endoscopy while treated with GLP-1 RAs. **Results**: GLP-1 RAs significantly increase the prevalence of clinically relevant RGC, despite prolonged fasting, with potential implications for airway protection and sedation safety. Although the risk of pulmonary aspiration remains low (≤0.15%), procedural delays, modifications, or cancellations can occur in up to 30% of cases without adapted protocols. Several professional societies (AGA, ASGE, AASLD) advocate for individualized management based on procedure type, symptomatology, treatment phase, and point-of-care gastric ultrasound (POCUS), in contrast to the systematic discontinuation recommended by the ASA. **Conclusions**: Effective management requires personalized fasting protocols, risk-based stratification, tailored anesthetic approaches, and interprofessional coordination. We propose a clinical decision algorithm and highlight the need for training in gastrointestinal pharmacology, POCUS, and airway management for endoscopists. Future priorities include prospective validation of clinical algorithms, safety outcome studies, and the development of intersocietal consensus guidelines.

## 1. Clinical and Pharmacological Introduction

Glucagon-like peptide-1 receptor agonists (GLP-1 RAs)—along with emerging dual and triple incretin co-agonists—have transformed the therapeutic landscape of type 2 diabetes mellitus (T2DM) and obesity, achieving sustained weight loss of 15–20%, enhanced glycemic control, and cardiovascular protection, as demonstrated in pivotal trials of once-weekly semaglutide and tirzepatide [1,2].

In addition to their incretin-mediated insulinotropic and glucagonostatic effects, GLP-1 RAs exert potent anorexigenic actions via central pathways (hypothalamus and brainstem) and peripherally attenuate gastric motility in a robust, dose-dependent manner [3,4]. This inhibition of gastric emptying (quantified through scintigraphy, stable-isotope breath testing, and capsule endoscopy) translates into a 30–40% prolongation in the half-time of solid gastric evacuation compared to placebo [5,6]. With long-acting weekly formulations, the prokinetic impairment persists for several days, such that omission of a single dose may not fully normalize gastric transit [2,7]. Contemporary meta-analyses estimate an absolute delay of 36 min (95% CI, 28–44) in gastric emptying at 4 h and a five- to sixfold increase in the odds of clinically relevant residual gastric content (RGC) exceeding 1.5 mL·kg^−1^ before elective diagnostic procedures [8,9,10].

Given their rapid uptake in clinical practice, a growing proportion of patients present for endoscopy while initiating or up-titrating GLP-1 RAs, precisely the phase in which motility suppression peaks [11,12]. Observational studies and systematic reviews report RGC in 12–30% of cases [7,13,14,15], with aborted or modified procedures in up to 30% [16,17]. While aspiration pneumonia remains rare, pooled data suggest odds ratios ≤ 1.8 and absolute incidence rates < 0.2% [18,19,20,21]. Moreover, GLP-1 RAs are associated with approximately a twofold increase in the likelihood of inadequate bowel preparation, frequently necessitating repeat colonoscopies [22,23,24].

Professional society guidance remains inconsistent. In 2023, the American Society of Anesthesiologists (ASA) issued a consensus statement recommending systematic drug discontinuation (on the day of the procedure for daily formulations and at least 7 days in advance for weekly agents) to mitigate the risk of a “full-stomach” scenario [25]. In contrast, the American Gastroenterological Association (AGA) rapid clinical update [25], the forthcoming position statement by the American Society for Gastrointestinal Endoscopy, and recently published multidisciplinary European and North American consensus documents [26,27] endorse a more individualized approach: prolonged clear-liquid fasting, preprocedural symptom screening, and selective use of gastric ultrasound, rather than universal suspension of GLP-1 RAs.

This manuscript offers a critical appraisal of the drug–procedure interface, synthesizing emerging evidence and articulating a pragmatic clinical algorithm rooted in risk stratification, optimized fasting protocols, selective gastric ultrasonography, and close collaboration between endoscopists and anesthesiologists. In parallel, it identifies key areas of uncertainty—including the need for prospective comparative trials and cost-effectiveness evaluations—that must be addressed to establish a coherent, internationally applicable standard of care.

## 2. Material and Methods for the Review of the Existing Evidence

### 2.1. Review Design and Registration

We conducted a structured narrative review with systematic mapping of the literature spanning from January 2005 to May 2025. The protocol was prospectively registered in the PROSPERO database (CRD42023378645) on 1 July of 2025 and adhered to PRISMA-ScR methodological guidelines.

### 2.2. Search Strategy

Comprehensive searches were performed in MEDLINE (via PubMed), Embase, and the Cochrane Library, employing the Boolean syntax: (“endoscopy” OR “gastroscopy” OR “colonoscopy” OR “capsule”) AND (“GLP-1 receptor agonist” OR “semaglutide” OR “tirzepatide”) AND (“gastric emptying” OR “residual gastric content” OR “aspiration”). In addition, reference lists from key meta-analyses were manually screened to identify further eligible studies [8,9,28].

### 2.3. Eligibility Criteria

We included randomized controlled trials, observational studies, case series comprising ≥3 patients, systematic reviews, and formal clinical guidelines addressing any endoscopic procedure in adult patients treated with GLP-1 RAs. Exclusion criteria were applied to non-English/Spanish publications, congress abstracts lacking full-text availability, and studies conducted exclusively in pediatric populations.

### 2.4. Study Selection

Titles and abstracts were independently reviewed by two investigators, with discrepancies resolved by consensus discussion. Of the 312 records initially identified, 68 proceeded to full-text assessment, of which 50 met the predefined inclusion criteria. These comprised 20 primary studies, 11 systematic reviews or meta-analyses, and 19 guidelines or illustrative case reports [10,29].

### 2.5. Data Extraction

A standardized data collection form was used to extract key study characteristics, including design, sample size, GLP-1 RA type and dosing regimen, pre-endoscopy fasting or drug-withdrawal protocols, method for assessing gastric content, and reported clinical outcomes (e.g., aspiration events, aborted procedures, bowel preparation adequacy). Definitions for “clinically relevant residual gastric content” (>1.5 mL·kg^−1^) and “aborted procedure” were aligned with those employed in high-quality meta-analyses [8,23].

### 2.6. Quality and Bias Assessment

Observational studies were assessed using the Newcastle-Ottawa Scale, while systematic reviews were appraised with the AMSTAR-2 instrument. Overall methodological quality was rated as high or moderate in 44% of studies and moderate in an additional 36%. The most frequent sources of bias stemmed from variability in fasting protocols and the absence of blinding in gastric ultrasound assessments [5,15].

### 2.7. Data Synthesis

Quantitative findings were summarized using risk ratios with corresponding 95% confidence intervals, anchored to pooled estimates from the most robust meta-analyses available [10]. Where gastric volume data were reported as means, values were standardized to mL·kg^−1^ using study-reported average body weights to facilitate cross-study comparison.

### 2.8. Limitations of the Evidence Base

The evidence base remains constrained by a limited number of randomized trials and substantial heterogeneity in fasting and drug-withholding protocols, which precluded formal comparative meta-analysis. Nevertheless, the broad array of study designs, patient populations, and procedural settings provide a sufficiently comprehensive foundation to inform the clinical algorithm proposed in subsequent sections of this manuscript.

## 3. Evidence on Gastric Emptying Delay and Residual Gastric Content

### 3.1. Physiological Basis

The delay in gastric emptying induced by GLP-1 receptor agonists (GLP-1 RAs) arises from a coordinated modulation of gastrointestinal motility under central vagal control. This involves relaxation of the proximal stomach, suppression of antral peristalsis, and tonic contraction of the pyloric sphincter, all mediated via vagally driven neurohumoral pathways [3]. Dose–response studies in healthy volunteers receiving graded intravenous GLP-1 infusions have demonstrated a linear correlation between systemic drug concentrations and the prolongation of the lag phase of solid gastric emptying [4]. Long-acting formulations, such as once-weekly semaglutide or tirzepatide, amplify this effect beyond their nominal pharmacokinetic half-lives, due to sustained receptor occupancy and prolonged downstream signaling. This phenomenon is corroborated by scintigraphy data obtained 72 h post-dosing in a substudy of the STEP-1 obesity trial, confirming that delayed emptying persists well into the dosing interval [1].

### 3.2. Experimental and Imaging Data

A seminal crossover trial employing C^13^-octanoate breath testing documented a 37% prolongation in gastric emptying half-time (T½) following four weeks of semaglutide 1 mg, compared with placebo [5]. Similar results (delays in the range of 32–38%) were observed using wireless motility capsules in patients treated with tirzepatide, reinforcing the cross-molecule reproducibility of the effect [6]. Point-of-care gastric ultrasonography (POCUS) has emerged as a pragmatic surrogate for scintigraphy in the procedural setting. In a prospective anesthesia cohort, semaglutide users exhibited solid-phase gastric content in 70–90% of scans performed eight hours after a standard fasting period, compared with 10–20% among matched non-users [7]. Another prospective volunteer study evaluated antral cross-sectional area and found a clear dose–response gradient: patients on 0.5 mg weekly semaglutide retained a mean gastric volume of 1.8 ± 0.7 mL·kg^−1^, whereas those on 2.4 mg had volumes of 2.6 ± 0.8 mL·kg^−1^ [12].

### 3.3. Observational Clinical Evidence

Real-world endoscopy series further corroborate these findings. Residual gastric content (RGC) graded as ≥2 was identified in 24% of semaglutide users vs. 5% of non-users during routine diagnostic upper endoscopy [13]. Drug interruption for fewer than 48 h did not significantly alter this prevalence [14]. However, cessation for ≥10 days was associated with a marked reduction in RGC incidence (from 20% to 6%) without triggering glycemic destabilization [11]. In a focused ultrasound triage study including 180 patients with diabetes, a ≥7-day withdrawal of GLP-1 RAs reduced the rate of “risk stomach” scans to 5%, compared to 17% in those who continued therapy [30]. Notably, a weight management clinic following strict clear-liquid fasting protocols for 24 h observed no significant increase in RGC prevalence despite continued GLP-1 RA use [31].

### 3.4. Evidence from Systematic Reviews and Meta-Analyses

A comprehensive meta-analysis encompassing 20 studies and 207,708 procedures found that GLP-1 RA exposure increased the odds of detecting RGC ≥ grade 2 by a factor of 5.6 [8]. In an independent synthesis of 21 cohorts, Baig et al. reported a pooled RGC prevalence of 18.4% (95% CI, 14.1–23.1) and established a 4.9-fold increase in the likelihood of aborted gastroscopies among GLP-1 RA users [9]. Elkin’s patient-level meta-analysis, based on 466,373 individuals, confirmed a sixfold elevation in RGC risk and highlighted that heterogeneity in fasting duration and timing of the last dose accounted for 48% of the interstudy variability [10]. Importantly, Tarar et al. demonstrated that POCUS significantly outperforms fasting history alone in predicting aspiration risk, with an area under the curve (AUC) of 0.91 [28].

### 3.5. Modifying Factors

Several key variables influence both the extent and the clinical relevance of GLP-1 RA-induced gastric stasis, shaping patient-level risk and procedural planning. Among them, dose and formulation stand out: higher weekly regimens (particularly semaglutide at doses ≥ 1 mg and tirzepatide ≥ 10 mg) are consistently linked with disproportionately prolonged gastric emptying times, as shown in mechanistic and clinical studies [5]. The duration of therapy also plays a modifying role; although the inhibitory effect on motility tends to attenuate after 12 to 16 weeks, likely due to partial vagal adaptation, this modulation is incomplete, and baseline gastric kinetics are not fully restored [6]. Comorbid diabetic gastroparesis, particularly in the context of autonomic neuropathy, markedly amplifies the risk, tripling the odds of significant residual gastric content when compared with euglycemic individuals [32]. Lastly, the structure of the fasting regimen exerts a notable impact: a clear-liquid fast maintained for at least 24 h can reduce the prevalence of clinically significant RGC by half, even when the GLP-1 RA is not withdrawn [32], underscoring the importance of preparatory dietary strategy in mitigating procedural risks. These modifying variables and their implications are summarized in Table 1.

#### Clinical Bottom Line

Converging lines of evidence—from foundational physiological studies and advanced imaging modalities to robust meta-analyses—consistently demonstrate that GLP-1 receptor agonists exert a reproducible, dose-dependent, and clinically significant delay in gastric emptying. This pharmacologically induced stasis results in the accumulation of residual gastric contents that often exceed conventional aspiration risk thresholds, particularly within the early months of therapy, when more than 20% of patients exhibit detectable intragastric residues despite standard fasting protocols. Although prolonged drug cessation or strict adherence to extended clear-liquid fasting regimens can attenuate this phenomenon, it rarely resolves entirely. The biological plausibility, magnitude, and consistency of this effect provide a compelling rationale for incorporating tailored pre-endoscopic precautions—including prolonged dietary restriction and selective use of bedside gastric ultrasonography—into routine clinical practice. Such adaptations are not merely precautionary but are firmly grounded in pathophysiology and supported by high-grade empirical data, reinforcing the imperative to align procedural planning with the pharmacodynamic realities of incretin-based therapies.

## 4. Risk of Aspiration and Endoscopic Complications

Pulmonary aspiration represents the most feared adverse event among endoscopists confronted with the strikingly high prevalence of RGC in patients receiving GLP-1 RAs. Nevertheless, the best available evidence consistently demonstrates that clinically significant aspiration is rare. In a large multicenter cohort involving 19,656 elective gastroscopies, only four episodes of clinically evident aspiration were reported (an incidence of just 0.02%) with no statistically significant difference between GLP-1 RA users and non-users, despite a 60% increase in same-day cancellations due to visible intragastric residue [18]. This finding is echoed in the largest available perioperative meta-analysis, encompassing 466,373 procedures, where GLP-1 RA therapy increased the odds of detecting RGC sixfold, yet the pooled odds ratio for aspiration remained statistically non-significant at 1.08 [10]. Two dedicated endoscopy-focused meta-analyses confirmed this pattern of risk dissociation, reporting a fivefold increase in aborted or modified procedures but no demonstrable excess in aspiration events [8,9].

This apparent discrepancy (abundant RGC but infrequent aspiration) has important practical implications. A close review of published case reports reveals a consistent clinical phenotype in the rare events that do occur: recent dose escalation, severe obesity, and deep sedation without airway protection are often present. For instance, aspiration has been documented in a patient with morbid obesity undergoing bariatric assessment just 48 h after a dose of semaglutide, despite a 20 h fast [33]; in another case, frank aspiration occurred at induction after 18 h of fasting [35]; and computed-tomography-confirmed pneumonitis was observed following diagnostic esophagogastroduodenoscopy (EGD) in a third instance [35]. While such reports inform clinical caution, they must be interpreted within the broader context of extremely low absolute risk, as demonstrated by cohort-level data.

Reconciling these two narratives (frequent gastric stasis but rare aspiration) requires careful attention to risk modifiers. As summarized in Table 1 (see previous section), factors such as drug dose, duration of therapy, presence of diabetic gastroparesis, and type of endoscopic procedure play key roles. Higher weekly doses (e.g., ≥1 mg semaglutide or ≥10 mg tirzepatide) impair solid gastric emptying more significantly than daily liraglutide, while patients in the early phases of treatment (within the first 8–12 weeks before central vagal adaptation emerges) demonstrate the largest ultrasound-measured gastric volumes [5]. Co-morbid diabetic autonomic neuropathy approximately triples the likelihood of significant RGC [40]. Moreover, the risk–benefit calculus becomes especially delicate for therapeutic procedures performed under general anesthesia or deep sedation without secured airways, where the consequences of aspiration are far more serious [34].

Given that clinically apparent aspiration remains uncommon, the predominant clinical burden associated with GLP-1 RA use is logistical rather than pulmonary: same-day cancellations or conversions of procedures when food or thick secretions are visualized in the gastric lumen. Following the American Society of Anesthesiologists (ASA) advisory in 2023, a multicenter audit in the United States found that the cancellation rate for diagnostic EGDs rose from 4% to 13% [17]. Meta-analytical data estimate that, on average, one in every twenty GLP-1 RA users scheduled for endoscopy experiences an aborted procedure [16].

In response to this emerging challenge, current guidelines coalesce around a risk-stratified framework. Prolonged clear-liquid fasting (defined as ≥24 h) consistently reduces the prevalence of RGC to single-digit percentages in most elective contexts [35]. Selective application of POCUS then serves as a reliable discriminator, distinguishing truly “full” stomachs (those requiring rapid-sequence induction or procedural postponement) from the vast majority of patients who may safely proceed with standard monitored anesthesia [30]. In this paradigm, drug discontinuation is not routine but rather reserved for patients who meet all three risk criteria outlined in Section 7. This nuanced approach helps avoid the glycemic rebound and weight regain observed in several cohorts following abrupt one-week cessation of GLP-1 RAs [44].

Clinical bottom line: The literature portrays pulmonary aspiration as a low-frequency but high-impact event. A triad of extended fasting, selective gastric ultrasonography, and airway protection in high-risk scenarios offers a rational, evidence-based strategy to mitigate this residual hazard—while avoiding the considerable metabolic consequences of indiscriminate drug discontinuation.

## 5. Colonoscopy and Capsule Endoscopy

Current evidence indicates that the profound, dose-dependent inhibition of gastric motility elicited by GLP-1 RAs extends beyond the stomach, exerting measurable downstream effects on colonic preparation and capsule endoscopy performance.

### 5.1. From Gastric Motor Inhibition to Colonic Cleansing Failure

Seminal scintigraphy studies involving once-weekly semaglutide revealed not only a 34 min prolongation in gastric half-emptying time (T½) but also a marked delay in the delivery of radiolabeled chyme to the small intestine, strongly suggesting suppression of phase III migrating motor complexes [5]. Complementary in vitro investigations demonstrated that GLP-1 reduces jejunal contractility by approximately one-third [4], confirming that its inhibitory effects are not confined to the gastric compartment. Together, these findings provide a robust mechanistic rationale for the impaired distribution and peristaltic propulsion of polyethylene glycol (PEG)-based laxatives frequently observed in clinical practice.

### 5.2. Clinical Studies of Bowel Preparation Quality

Three large-scale observational studies consistently corroborate these pathophysiological insights. In a multicenter Israeli cohort encompassing 9752 colonoscopies, GLP-1 RA exposure independently doubled the likelihood of inadequate cleansing, defined as a Boston Bowel Preparation Score (BBPS) < 6, even after controlling for diabetes, age, and purgative volume [22]. Similarly, a North American single-center study involving 446 procedures reported a comparable excess risk (15.5% vs. 6.6%), which persisted despite the use of split-dose 4L PEG protocols [24]. A regional health system database comprising over 12,000 colonoscopies further showed that a 7-day semaglutide interruption failed to restore bowel preparation to baseline levels (adjusted OR 1.6) and was accompanied by modest, yet measurable, elevations in HbA1c and early weight regain [41]. Pooled analysis from eight cohort studies estimated that GLP-1 RA therapy increases the relative risk of inadequate cleansing by 80%, yielding a number needed to repeat (NNR) of just 14 colonoscopies [23]. These findings are summarized in Table 2.

### 5.3. Downstream Consequences

Missed lesions and repeat procedures: The clinical repercussions of suboptimal bowel preparation are far from trivial. In a real-world ambulatory network, patients on GLP-1 RAs were three times more likely to require repeat colonoscopy within 12 months compared to non-users (9.4% vs. 3.1%), with obvious implications for patient burden and healthcare system costs [45]. In a prospective study evaluating mucosal visibility, 23% of patients receiving semaglutide exhibited obscured views in the proximal colon. This finding not only extended withdrawal times but also resulted in a significant reduction in adenoma detection rates when compared to matched controls [34].

### 5.4. Optimizing Preparation Without Universal Drug Suspension

Encouragingly, intensified preparation protocols may obviate the need for routine drug withdrawal. In a pragmatic series involving bariatric patients, a regimen combining a 24 h clear-liquid diet with split-dose 4L PEG achieved adequate bowel cleansing in 92% of GLP-1 RA users who continued their medication uninterrupted, with no observed glycemic destabilization [40]. These findings have informed recent multisociety recommendations that prioritize enhanced purgative strategies and extended liquid fasting over systematic pharmacological cessation. A ≥7-day hold is now reserved for a narrowly defined subgroup: patients with prior failed preparations despite intensified regimens or those exhibiting overt symptoms of upper gastrointestinal dysmotility [36].

### 5.5. Capsule Endoscopy

Transit kinetics and completion rates: Wireless capsule endoscopy offers additional granularity regarding gastrointestinal transit dynamics under GLP-1 RA treatment. Prospective monitoring has shown that median gastric transit time (GTT) increases from 34 to 78 min in patients on weekly semaglutide. Capsule completion rates consequently fall from 92% to 87%, with most incomplete studies occurring in individuals receiving high doses during the early phases of therapy [46]. Small-bowel transit is also impaired: motility capsule recordings in recipients of 15 mg tirzepatide revealed a mean delay of 42 min compared with baseline [31]. Although no confirmed cases of true capsule retention have been directly attributed to GLP-1 RAs, delayed gastric exit necessitated overnight real-time verification in 8% of cases and endoscopic duodenal deployment in two [28]. A survey of European endoscopists underscores the procedural variability and clinical uncertainty surrounding capsule protocols, with marked differences in the use of prokinetics and airway protection strategies [41].

### 5.6. Practical Recommendations

Preparation should be tailored to individual risk. For the majority of patients, a 24 h clear-liquid diet combined with high-volume split-dose PEG is sufficient. In early-phase or high-dose users, a 48 h low-residue diet supplemented with adjunctive bisacodyl may further improve outcomes. Given the limited efficacy and potential metabolic consequences of brief drug interruptions, discontinuation for ≥7 days should be restricted to cases of proven preparation failure despite intensified regimens or to those manifesting florid upper gastrointestinal symptoms.

For capsule endoscopy, real-time viewers should verify gastric exit before committing to interpretation. If GTT exceeds 60 min, administration of 10 mg intravenous metoclopramide or direct endoscopic duodenal placement is advised to ensure procedural completion.

Finally, there remains an urgent need for randomized trials comparing advanced bowel preparation strategies vs. temporary GLP-1 RA cessation, as well as formal cost-effectiveness analyses of real-time capsule monitoring in this growing patient population.

Clinical bottom line: The cumulative evidence unequivocally demonstrates that GLP-1 receptor agonists impair colonic preparation and delay capsule transit, increasing the probability of incomplete examinations and necessitating repeat procedures. While these effects are clinically meaningful, they can be substantially mitigated through intensified bowel cleansing protocols and real-time monitoring. Prolonged drug withdrawal should be reserved for clearly delineated high-risk scenarios or for patients in whom optimized preparatory strategies have failed.

## 6. Summary of Clinical Guidelines and Positions

Since mid-2023, at least five authoritative guidance documents have sought to delineate best practices for managing GLP-1 receptor agonists (GLP-1 RAs) in the context of gastrointestinal endoscopy. Although they diverge in tone (spanning from categorical suspension to a more nuanced, patient-centered approach) their composite message forms a coherent clinical trajectory that underpins the risk-adapted algorithm described in Section 7.

### 6.1. The Precautionary Era: American Society of Anesthesiologists (ASA)

The debate was catalyzed by the Consensus-Based Guidance issued by the ASA regarding perioperative management of patients receiving GLP-1 RAs [38]. Drawing primarily on isolated case reports and expert opinion, the ASA advised withholding daily GLP-1 RA formulations on the morning of the procedure and suspending weekly agents for a full seven days prior to elective interventions. Despite the low evidence grade, the recommendation rapidly permeated into endoscopic practice, triggering a notable uptick in same-day cancellations and reschedulings [17].

### 6.2. Gastroenterology Push-Back: AGA Rapid Update

In direct response, the American Gastroenterological Association (AGA) released a concise “rapid clinical practice update” within five months, challenging the ASA’s stance as unsupported by robust data and logistically unfeasible for routine care [25]. The AGA instead advocated for a 24 h clear-liquid fast, reserving GLP-1 RA cessation for patients with persistent upper gastrointestinal symptoms. Importantly, the document introduced POCUS as a discriminative triage modality, a recommendation subsequently adopted by anesthesia societies.

### 6.3. Procedural Nuance: American Society for Gastrointestinal Endoscopy (ASGE)

The ASGE, in its 2025 position statement, endorsed the AGA’s risk-stratified philosophy but added procedural granularity. It distinguished low-risk diagnostic procedures (such as screening EGDs and colonoscopies under moderate sedation) from higher-risk therapeutic interventions, including EUS, ERCP, and endoscopic sleeve gastroplasty (ESG). For the former, a 24 h clear-liquid fast suffices in asymptomatic patients; for the latter, airway protection or deferral is warranted if POCUS reveals solid gastric contents.

### 6.4. Multidisciplinary Convergence: Kindel and Colleagues

A multisociety collaboration (comprising the AGA, ASA, ASGE, American Association for the Study of Liver Diseases (AASLD), and Society for Bariatric Endoscopy) crystallized these evolving practices into a unified, algorithmic consensus [36]. This framework codifies the now widely cited “triple screen” model, which entails: (i) symptom assessment, (ii) stratification by procedural risk, and (iii) selective POCUS when ambiguity remains regarding gastric status.

### 6.5. European and North American Anesthesia Perspectives

In the United Kingdom, the Royal College of Anesthetists, the Association of Anesthetists, and the British Society of Gastroenterology jointly issued guidance favoring continued GLP-1 RA use, provided it is coupled with extended clear-liquid fasting and access to preprocedural gastric ultrasonography [26]. Drug withdrawal for ≥7 days is recommended only in the setting of high-risk airway procedures combined with unresolved upper-GI symptoms. A Delphi-based consensus from SPAQI in the United States reached similar conclusions, underscoring the importance of institutional training in POCUS and shared decision-making frameworks [27].

### 6.6. Key Areas of Agreement and Divergence

Across these documents, there is broad consensus on several foundational principles: (1) aspiration events are rare, and universal drug suspension lacks evidentiary support. (2) Clear-liquid fasting of ≥24 h substantially reduces the prevalence of residual gastric content. (3) POCUS is a valuable tool for procedural triage where local expertise is available.

The primary axis of divergence concerns the threshold for drug discontinuation. While the ASA maintains a precautionary blanket hold of seven days for weekly agents, most gastroenterology and anesthesia bodies now limit cessation to high-risk cases: those with active symptoms or ultrasound-documented gastric retention undergoing complex interventions.

#### Clinical Bottom Line

**Figure 1:**
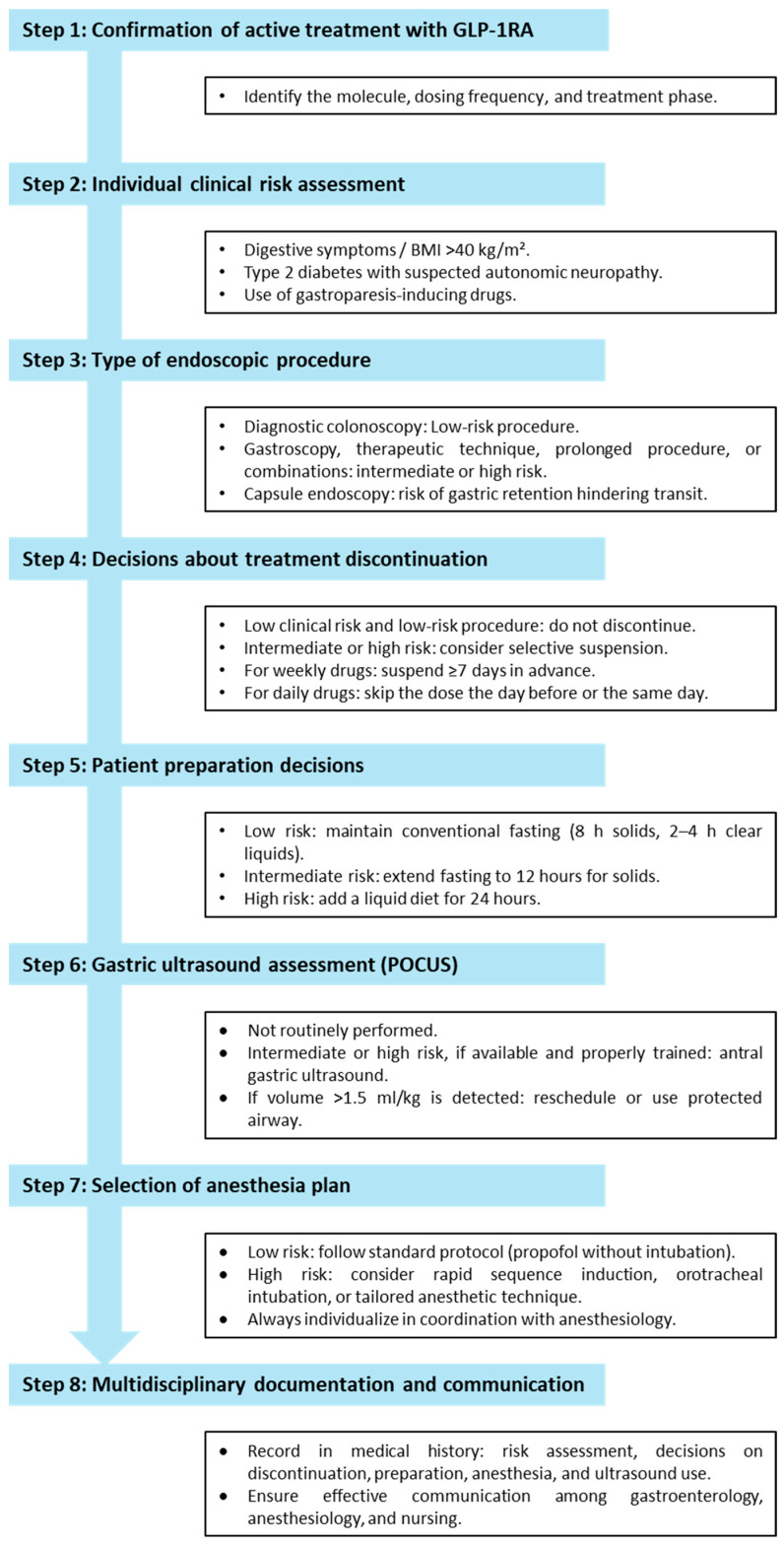
Suggested integrated Clinical Decision Algorithm (Sequential and Operational Version). GLP-1RA: glucagon-like peptide-1 receptor agonist; BMI: body mass index; POCUS: point-of-care gastric ultrasound.

**Figure 2:**
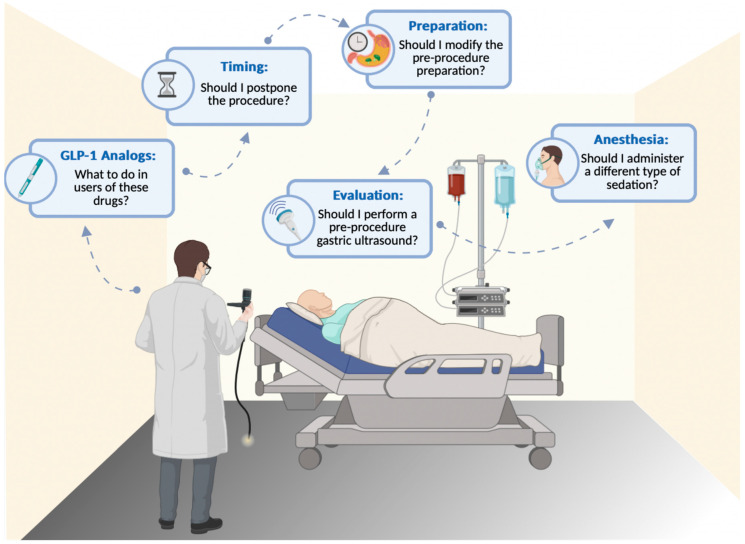
Suggested Protocol for Managing GLP-1 RA Treatment Around Endoscopic Procedures. GLP-1 RA: glucagon-like peptide-1 receptor agonist.

Taken collectively, these positions endorse a risk-adapted paradigm that balances safety with feasibility, minimizing unnecessary cancellations while preserving the substantial metabolic benefits conferred by GLP-1 RA therapy. The synthesis above, supported by the critical considerations in Table 3 and the integrative flowchart in Figure 1 and Figure 2, equips endoscopy teams to implement these recommendations pragmatically. Tailoring practice to institutional infrastructure (particularly regarding the availability of gastric ultrasound and anesthesia support) allows clinicians to maintain procedural safety without incurring the metabolic and logistical costs associated with indiscriminate drug withdrawal.

## 7. Integrated Clinical Decision Algorithm

The proposed clinical algorithm commences by identifying the specific GLP-1 receptor agonist in use, its current dosing regimen, and the interval since the last administration. This step is pivotal, as long-acting formulations such as semaglutide and tirzepatide exert a sustained inhibitory effect on gastric transit that may persist for seven days or longer [2], particularly at higher doses, which have been shown to significantly prolong the half-emptying time of solids [5]. Immediately following this, a brief symptom screening is undertaken. Patients reporting nausea, early satiety, or postprandial fullness are approximately three times more likely to harbor clinically significant residual gastric content [40], rendering them at elevated risk for aspiration or procedural modification.

The subsequent step requires the endoscopist to assess the intrinsic airway risk of the planned intervention. Procedures such as endoscopic ultrasound (EUS), endoscopic retrograde cholangiopancreatography (ERCP), or endoscopic sleeve gastroplasty (ESG)—particularly when performed under general anesthesia—pose heightened consequences in the event of regurgitation or aspiration. Notably, European audit data have revealed considerable heterogeneity in the implementation of airway protection for such high-risk procedures [41], underscoring the need for structured decision making.

Regardless of procedural tier, all patients are instructed to undergo a baseline clear-liquid fast of no less than 24 h. In cases involving recent exposure to high-dose therapy or the presence of upper gastrointestinal symptoms, this is prudently extended to 48 h of solid-food abstinence. This recommendation is grounded in prospective ultrasound data indicating that up to 90% of semaglutide-treated individuals still display solid gastric content eight hours after a conventional fast [7], highlighting the inadequacy of standard fasting protocols in this population.

POCUS is then employed selectively, primarily in symptomatic patients or when high-risk procedures coincide with recent drug administration. With a validated sensitivity of 86% and specificity of 94% for identifying a “full” stomach [30], POCUS offers critical discrimination. When imaging reveals a grade 0 or grade 1 antrum, the procedure may safely proceed under routine monitored anesthesia. In contrast, the presence of solid contents or an antral cross-sectional area suggestive of volumes exceeding 100 mL necessitates the presumption of a non-fasted state, thereby triggering either a procedural delay or induction with rapid-sequence intubation using a cuffed endotracheal tube. These measures are consistent with both British [26] and North American [27] multidisciplinary guidance. In scenarios where ultrasound is unavailable, clinicians should default to the same aspiration-prevention strategies if recent GLP-1 RA use, suggestive symptoms, and procedural risk co-exist.

Pharmacological withdrawal is reserved for a narrowly defined triad of criteria: recent drug administration (within seven days), a planned high-risk airway intervention, and either positive symptomatology or sonographic evidence of solids. Outside of this constellation, therapy continuation is preferred, as aspiration events remain exceptionally rare (0.02% across 19,656 elective EGDs) [18], while interruption of GLP-1 RA treatment may precipitate glycemic destabilization or early weight rebound [44]. In cases where endotracheal intubation is required, recent reports of frank aspiration despite conventional fasting underscore the necessity of employing rapid-sequence induction with full airway protection [35].

For colonoscopy, data clearly favor an approach centered on enhanced bowel preparation rather than routine drug discontinuation. A combined regimen consisting of a 24 h clear-liquid diet, split 4 L polyethylene glycol, and adjunctive bisacodyl has been shown to achieve adequate cleansing in over 90% of patients who continue their GLP-1 RA therapy uninterrupted [40]. In contrast, a seven-day drug hold has demonstrated limited efficacy in restoring bowel preparation quality and carries the added risk of metabolic deterioration [23]. As such, intensified purgative regimens remain the first-line strategy for this population [36].

Capsule endoscopy protocols should incorporate real-time gastric transit monitoring. Capsules that fail to exit the stomach within 60 min may respond to 10 mg intravenous metoclopramide or, alternatively, be advanced directly into the duodenum via endoscopy [42]. This adaptive strategy obviates the need to routinely exclude GLP-1 RA users from capsule procedures, particularly as no cases of true retention attributable to this drug class have been documented to date [28].

Therapy resumption should be tailored to the clinical context. In low-risk procedures with no significant findings, GLP-1 RA treatment may be reinstated once the patient resumes oral intake. However, for individuals with documented significant residual gastric content or aspiration, therapy should be reinitiated only after tolerance of solid food has been demonstrated and metabolic stability has been restored. This cautious approach aligns with the pragmatic guidance articulated by the ASA [38].

Clinical bottom line: This sequential framework, beginning with documentation of GLP-1 RA exposure, followed by symptom assessment, procedural risk stratification, implementation of extended clear-liquid fasting, selective use of gastric ultrasonography, and reserving drug withdrawal for rigorously defined indications, harmonizes current pathophysiological understanding with the best available evidence and major society recommendations. In doing so, it offers a clinically practical yet safety-conscious pathway that minimizes both the low but real risk of aspiration and the substantial metabolic repercussions of interrupting an increasingly essential therapy for obesity and type 2 diabetes.

## 8. Key Clinical Questions and Evidence-Based Recommendations

**Q1:**  *Should all patients on GLP-1 receptor agonists discontinue therapy prior to diagnostic upper endoscopy?*

Evidence: The prevalence of residual gastric content (RGC) is markedly elevated among GLP-1 RA users—observed in 24% compared with 5% in matched controls [13], with a pooled odds ratio of 5.6 in meta-analysis [8]. Nevertheless, the incidence of aspiration remains exceedingly low, with just 0.02% recorded among 19,656 elective EGDs [18,39].

Recommendation: Routine drug withdrawal is not justified by the data. Instead, implement a 24 h clear-liquid fast and reserve discontinuation for high-risk procedures when patients are symptomatic or gastric ultrasound reveals solid contents, ideally with a minimum 7-day drug hold under such conditions.

**Q2:**  *What fasting regimen is appropriate when GLP-1 RA therapy is continued?*

Evidence: A 24 h clear-liquid regimen successfully reduced RGC prevalence to 8% and enabled the safe continuation of GLP-1 RA therapy in 92% of bariatric patients [40]. By contrast, ultrasound data show that standard fasting of eight hours leaves significant residuals in up to 90% of semaglutide users [7].

Recommendation: A minimum of 24 h on clear liquids should be enforced prior to any sedated upper-GI procedure; this interval should be extended to 48 h in the context of recent high-dose administration or active upper-GI symptoms.

**Q3:**  *When is POCUS indicated?*

Evidence: POCUS demonstrates robust diagnostic performance, with 86% sensitivity and 94% specificity for detecting a “full” stomach [32], and has been shown to alter procedural management in approximately one-third of prospective cases [12].

Recommendation: POCUS should be selectively employed in cases involving recent weekly GLP-1 RA intake (≤7 days) and/or upper-GI symptoms, especially when high-risk airway procedures are planned. A grade 0 or 1 antrum permits the procedure to proceed under standard monitored anesthesia, while grade 2 findings or solid content ≥ 100 mL warrant management as a full-stomach scenario.

**Q4:**  *Which endoscopic procedures require airway protection?*

Evidence: All reported instances of aspiration in GLP-1 RA users occurred under deep sedation without airway protection [33,35]. Despite this, a European survey revealed that only 38% of EUS and ERCP procedures are routinely performed with intubation [45].

Recommendation: Endotracheal intubation or rapid-sequence induction should be used for high-risk procedures such as EUS, ERCP, bariatric endoscopy, or any sedated intervention where sonographic RGC is present. Routine monitored anesthesia is acceptable for low- to moderate-risk EGDs when POCUS findings are negative.

**Q5:**  *How should bowel preparation for colonoscopy be adapted in GLP-1 RA users?*

Evidence: GLP-1 RA therapy is associated with an 80% increase in the risk of inadequate bowel cleansing (relative risk 1.8) [23], and a seven-day drug interruption often fails to restore normal preparation [44].

Recommendation: The primary strategy should focus on enhancing the purgative regimen: split-dose 4L polyethylene glycol, with or without bisacodyl, combined with a 24 h clear-liquid diet. Temporary drug withdrawal should be considered only after documented failure of this intensified approach.

**Q6:**  *What constitutes best practice for capsule endoscopy in these patients?*

Evidence: Gastric transit time extends from a median of 34 min to 78 min under weekly semaglutide, with a modest decline in completion rates to 87% [46]. While capsule retention has not been attributed to GLP-1 RA therapy, delayed gastric exit required intervention in approximately 8% of cases [28].

Recommendation: Real-time capsule monitoring is essential. If gastric transit exceeds 60 min, administer 10 mg IV metoclopramide; if retention persists at 90 min, consider endoscopic duodenal deployment. Routine preprocedural drug discontinuation is unwarranted.

**Q7:**  *What are the metabolic implications of drug withdrawal, and how should they be balanced against procedural risk?*

Evidence: Discontinuation of GLP-1 RAs for seven days or more has been associated with a mean HbA1c rebound of 0.3% and an average weight regain of 0.7 kg [34]. Meta-analyses, however, have not demonstrated any meaningful increase in aspiration risk when therapy is continued [10].

Recommendation: Continuation of GLP-1 RA therapy should be the default strategy whenever fasting and ultrasound findings are reassuring. Discontinuation should be restricted to the small subset of patients with high-risk procedures, recent high-dose drug exposure, and either positive ultrasound findings or significant symptoms.

### Clinical Bottom Line

A carefully stratified approach—rather than indiscriminate cessation—offers optimal protection against the rare but serious risk of aspiration, while preserving the metabolic and cardiovascular benefits of uninterrupted GLP-1 RA therapy. The triad of extended clear-liquid fasting, selective gastric ultrasonography, and procedure-specific airway management constitutes a robust and evidence-based safety framework.

## 9. Need for Training and Changes in Endoscopic Practice

With one in every four elective endoscopy patients soon expected to be receiving GLP-1 RAs, this epidemiological reality mandates a parallel evolution in workforce competencies, procedural logistics, and educational priorities within modern endoscopy units.

### 9.1. Competency-Based Curricula

Contemporary competency-based medical education in endoscopy has traditionally emphasized lesion detection, resection techniques, and adherence to quality metrics [46]. However, revised ASGE guidance now expands this scope, incorporating “pharmacology-related risk mitigation” as a core domain. Trainees are expected to demonstrate command of fasting algorithms, drug-withdrawal thresholds, and airway protection strategies prior to unsupervised practice [47]. Echoing this shift, the ESGE’s quality framework now explicitly includes medication-specific preparation protocols as emerging benchmarks of procedural excellence, alongside established indicators such as adenoma detection rate [43].

### 9.2. Simulation and Team Rehearsal

A robust body of evidence confirms that high-fidelity simulation not only accelerates technical skill acquisition but also ensures reliable transfer to real-world clinical performance [48]. Within the GLP-1 RA context, simulation modules should reflect contemporary challenges, including: (a) intraprocedural detection of unexpected solid gastric residue, (b) rapid-sequence induction protocols under time pressure, and (c) troubleshooting of delayed capsule transit during wireless endoscopy.

A prospective intervention employing a bespoke gastric-content and airway manikin improved first-pass intubation rates from 68% to 94% among endoscopy nurses without prior anesthetic experience, illustrating the transformative impact of targeted simulation [49].

### 9.3. Training in Bedside Gastric Ultrasound (POCUS)

Given the pivotal role of point-of-care gastric ultrasonography within the proposed risk-stratification algorithm (Section 7), structured and replicable training pathways are essential. A recently published schema proposes a four-phase progression (didactic instruction, supervised scanning of healthy volunteers, supervised application in high-risk patients, and summative assessment via OSCE) that leads to reliable competence after approximately 25 mentored examinations [50]. Embedding this trajectory within endoscopy fellowships would allow practitioners to independently perform and interpret POCUS, reducing dependency on anesthesia services and enhancing workflow resilience.

### 9.4. Sedation, Airway Management, and Interdisciplinary Communication

The ASGE’s 2025 sedation curriculum update explicitly addresses incretin-based therapies, calling for expanded proficiency in risk triage and airway decision making. Endoscopists should be capable of initiating rapid-sequence induction when confronted with a full stomach, particularly in the absence of anesthetic support. Crew-resource management (CRM) training (adapted from aviation to clinical settings) has been shown to reduce critical-event response times by up to 40% during simulated aspiration scenarios, reinforcing the value of multidisciplinary rehearsal as routine, not exceptional.

### 9.5. Patient Education and Digital Engagement Tools

One of the most frequent contributors to failed bowel preparation in GLP-1 RA users is suboptimal communication regarding the 24 h clear-liquid regimen. Digital solutions have shown promise: interactive smartphone applications delivering timed notifications and visual instructions (e.g., animated GIFs) improved Boston Bowel Preparation Scores by a full point in a randomized pilot trial [24]. Future refinements could include algorithm-driven alerts tailored to GLP-1 RA pharmacokinetics (for instance, “Your last semaglutide dose was 4 days ago; please contact the endoscopy unit”) thereby minimizing last-minute cancellations.

### 9.6. Workflow and Systems Redesign

Endoscopy scheduling systems should be reconfigured to automatically flag patients on weekly GLP-1 RAs during the booking phase, triggering longer procedural slots or proactive ultrasound assessment for high-risk cases. Implementation of such features in a two-center quality improvement initiative reduced same-day cancellations by 27% [45], underscoring the operational impact of simple yet targeted digital interventions.

### 9.7. Research and Accreditation Implications

As these changes gain traction, accreditation bodies may soon mandate documentation of training in pharmacology-driven fasting protocols and POCUS application. While head-to-head trials comparing simulation-enhanced curricula to traditional apprenticeship models are still lacking, early evidence suggests significant gains in both procedural safety and operational efficiency.

#### Clinical Bottom Line

Adapting endoscopic practice to the widespread use of GLP-1 receptor agonists entails more than publishing algorithms. It requires the deliberate redesign of training pathways, the strategic integration of simulation infrastructure, and the adoption of digital tools that support both patients and providers. By embedding these competencies into current education frameworks, endoscopy units can mitigate aspiration risk, maintain metabolic stability, and ensure procedural quality without sacrificing throughput.

## 10. Conclusions

GLP-1 RAs have profoundly transformed the therapeutic landscape of metabolic medicine, becoming integral to the management of type 2 diabetes and obesity. As their clinical use expands, so too does the proportion of patients presenting for endoscopic procedures while receiving these agents. The cumulative body of evidence reviewed herein leaves little room for ambiguity: GLP-1 RAs induce a consistent, dose-dependent delay in gastric emptying—raising solid-phase T½ by approximately one-third and increasing the likelihood of RGC by a factor of five to six [5,8].

Paradoxically, this striking physiological disruption does not manifest in a proportionate rise in clinically significant pulmonary aspiration; indeed, the observed increase remains marginal and statistically non-significant in the largest available series [18]. Thus, while the pathophysiological signal is robust, the primary clinical burden emerges elsewhere (in elevated rates of procedure cancellation, suboptimal bowel preparation, and prolonged capsule transit), each of which carries practical and logistical consequences for endoscopy services. These implications are synthesized in Table 4.

In light of these findings, a coherent, evidence-based strategy can be articulated around three interlocking pillars:

(1) The adoption of extended clear-liquid fasting (≥24 h), complemented by selective point-of-care gastric ultrasonography in patients reporting upper gastrointestinal symptoms or undergoing high-risk procedures that involve airway sharing. This dual-pronged approach enables the safe continuation of GLP-1 RAs in the vast majority of cases while reserving drug cessation (≥7 days) for a narrowly defined subset, namely, those with recent high-dose exposure, significant residual content on ultrasound, or florid symptoms suggestive of impaired gastric motility.
(2) The prioritization of intensified bowel preparation regimens (particularly split-dose polyethylene glycol solutions in high volume, optionally augmented with stimulant laxatives) over indiscriminate drug withdrawal. Such regimens have been shown to restore colonic cleansing efficacy without compromising metabolic control, thereby safeguarding colonoscopy quality while avoiding glycemic rebound or weight regain [23].
(3) The implementation of targeted training and service reconfiguration. This includes the formal integration of pharmacology-specific fasting algorithms, structured ultrasound training, and rapid-sequence induction competencies into endoscopic curricula, alongside the deployment of digital engagement tools designed to support patient adherence to prolonged fasting protocols. These structural adaptations not only enhance patient safety but also sustain throughput and operational efficiency.

Taken together, these pillars forge a pragmatic and clinically defensible pathway that reconciles endoscopic safety with uninterrupted metabolic benefit. Moreover, they align institutional practice with the converging recommendations of multiple learned societies and position endoscopy units to navigate the growing influx of GLP-1 RA users without compromising procedural integrity or performance metrics.

Looking ahead, future research should prioritize the generation of prospective, randomized data comparing extended fasting alone with formal drug discontinuation strategies. Cost-effectiveness analyses of routine preprocedural ultrasonography and simulation-based training interventions are likewise needed to strengthen the evidence base. These investigations will refine the algorithm proposed in this review and move the field towards a standard of care that is both evidence-driven and centered on the needs of the modern metabolic patient.

## Figures and Tables

**Table 1:** Main arguments about the suspension of GLP-1 RA before endoscopy.

Arguments for SUSPENDING or Delaying	References	Arguments for NOT Suspending or Delaying	References
↑ Gastric retention (>15%) demonstrated by EGD or ultrasound	[1,3,4,5,6,7,8,9,12,13,14,15,18,21,22,23,24,28,32,33,34]	Absolute risk of aspiration low or not increased in cohorts/meta-analyses	[10,18,19,20,29,35,36]
Cases of aspiration/regurgitation despite standard fasting	[7,13,34,36,37]	Liquid fasting 18–24 h + ultrasound allows for continuing	[25,26,38,39]
Meta-analysis: ↑ EGD abortions or colonic malpreparation	[5,8,9,16,23,28,39]	Stopping ≥ 7–10 days can uncontrol blood sugar/weight and delay attention	[38,40,41,42]
High-risk procedures (EUS, deep sedation without protected airway)	[26,39,42]	Real series show safety with continuous AR	[33,36,40]
Physiological basis: gastric emptying ↓ 30–40%	[1,3,4,6]	Consensus and additional research needed	[26,27,43]

**Table 2:** Quality of Bowel Preparation.

Study (Reference)	Design/n	Key Finding
[22]	Multicenter case–control, 9752 colonoscopies	GLP-1 RA doubled inadequate cleansing (Boston Score < 6); risk independent of diabetes and PEG volume.
[24]	Single-center cohort, n = 446	Poor prep 15.5% vs. 6.6% (controls); split-dose 4 L PEG mitigated, but did not abolish, the gap.
[44]	Regional database, >12,000 exams	Seven-day semaglutide hold failed to normalize prep (OR 1.6); HbA1c rebounded 0.3%.
[23]	Meta-analysis, 8 studies	Pooled RR 1.8 (95% CI 1.4–2.2) for inadequate cleansing; NNT to repeat colonoscopy ≈ 14.

**Table 3:** Modifiers of Aspiration Risk.

Modifier	Evidence	Clinical Implication
Dose/Potency	Higher weekly doses (≥ 1 mg semaglutide or ≥ 10 mg tirzepatide) show longer gastric half-emptying times [5].	Consider longer fasting or ultrasound if high doses given within 7 days.
Duration of therapy	Gastric adaptation after 12–16 weeks reduces, but does not normalize, RGC rates [6].	Early-phase users are higher risk.
Co-morbid gastroparesis	Diabetic autonomic neuropathy triples RGC odds [32].	Symptomatic diabetics warrant ultrasound or extended fasting.
Procedure type and sedation	EUS or ERCP under general anesthesia carry greater aspiration potential; European survey shows only 38% routinely protect airway [37].	Prefer endotracheal intubation for high-risk procedures in GLP-1 RA users.

**Table 4:** Main Clinical Implications of GLP-1RA Use in Digestive Endoscopy.

GLP-1RA-induced delayed gastric emptying is a reproducible physiological finding with direct implications for bronchoaspiration risk during sedated procedures. The prevalence of residual gastric content is significantly higher in patients treated with GLP-1 RAs, with documented impact on procedure cancellation or modification. The absolute clinical aspiration risk is low but not negligible, especially in early treatment phases or patients with active digestive symptoms. Systematic treatment suspension lacks solid support and may adversely affect metabolic control. Individualized evaluation integrating symptoms, treatment phase, co-morbidities, and objective tools like gastric ultrasound (POCUS) is recommended. Specialized training in sedation and aspiration risk management by gastroenterologists is emphasized.

## Abbreviations

The following abbreviations are used in this manuscript:

AASLD	American Association for the Study of Liver Diseases
AGA	American Gastroenterological Association
ASA	American Society of Anesthesiologists
ASGE	American Society for Gastrointestinal Endoscopy
BMI	Body mass index
ESGE	European Society for Gastrointestinal Endoscopy
GLP-1 RAs	GLP-1 receptor agonists
NNH	Number needed to harm
NNT	Number needed to treat
POCUS	Point-of-care ultrasound
RGC	Residual gastric content
T2DM	Type 2 diabetes mellitus
